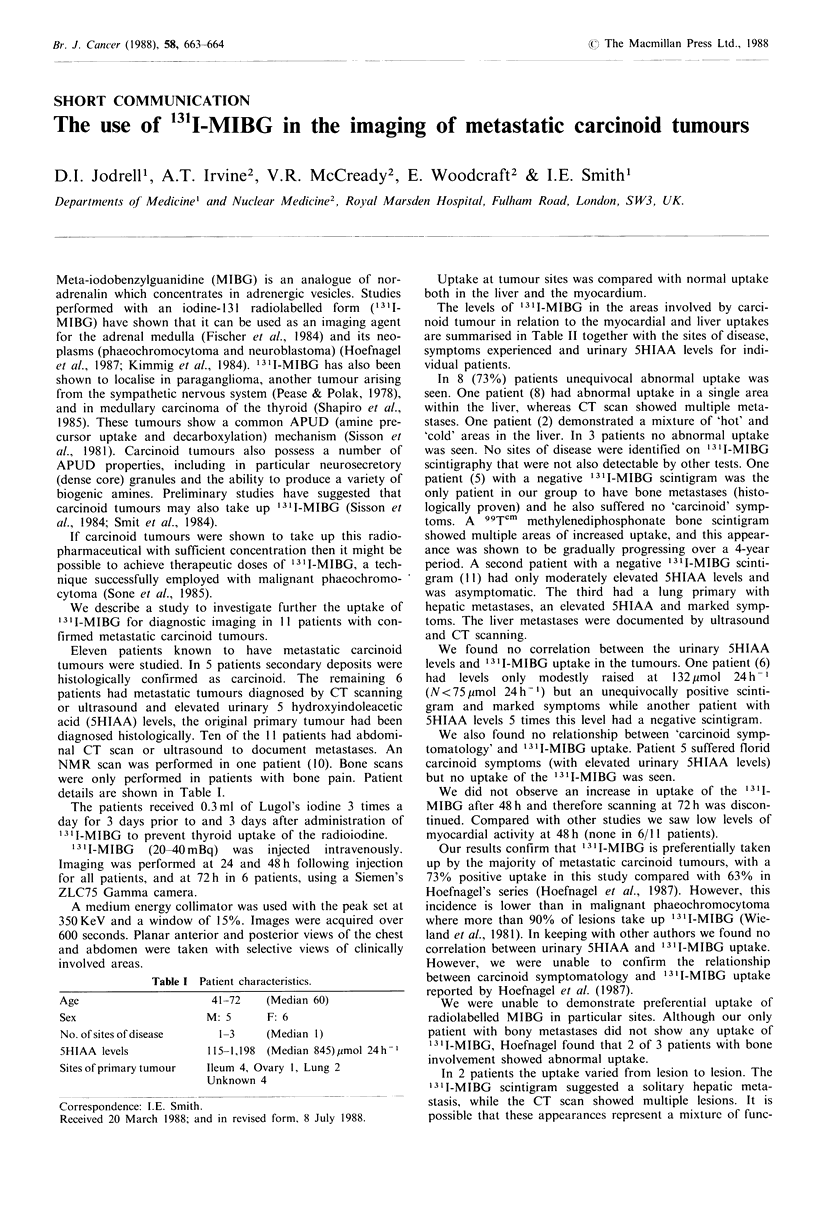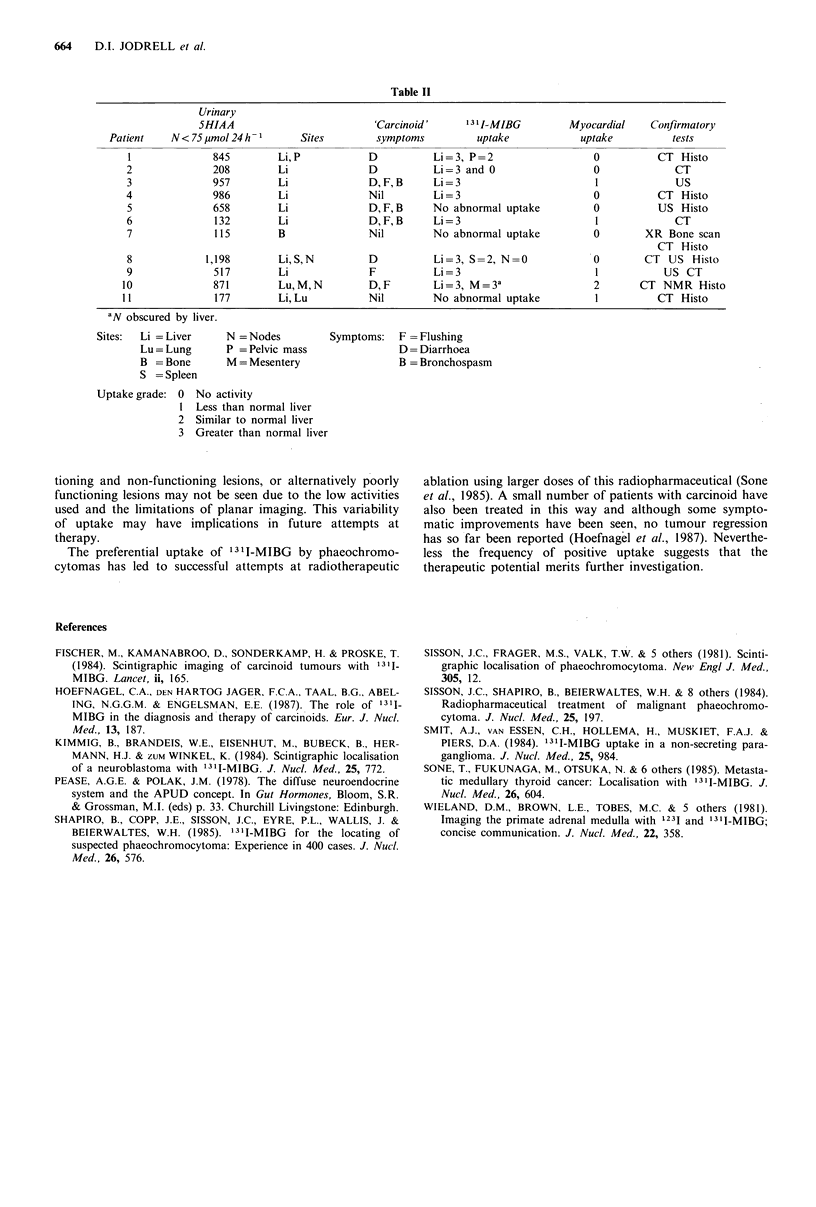# The use of 131I-MIBG in the imaging of metastatic carcinoid tumours.

**DOI:** 10.1038/bjc.1988.281

**Published:** 1988-11

**Authors:** D. I. Jodrell, A. T. Irvine, V. R. McCready, E. Woodcraft, I. E. Smith

**Affiliations:** Department of Medicine, Royal Marsden Hospital, London, UK.


Br. J. Cancer (1988), 58, 663-664 © The Macmillan Press Ltd., 1988

SHORT COMMUNICATION

### The use of $^{131}$I-MIBG in the imaging of metastatic carcinoid tumours

D.I. Jodrell[1], A.T. Irvine[2], V.R. McCready[2], E. Woodcraft[2] & I.E. Smith[1]

*Departments of Medicine[1] and Nuclear Medicine[2], Royal Marsden Hospital, Fulham Road, London, SW3, UK.*

Meta-iodobenzylguanidine (MIBG) is an analogue of noradrenalin which concentrates in adrenergic vesicles. Studies performed with an iodine-131 radiolabelled form ($^{131}$I-MIBG) have shown that it can be used as an imaging agent for the adrenal medulla (Fischer *et al.*, 1984) and its neoplasms (phaeochromocytoma and neuroblastoma) (Hoefnagel *et al.*, 1987; Kimmig *et al.*, 1984). $^{131}$I-MIBG has also been shown to localise in paraganglioma, another tumour arising from the sympathetic nervous system (Pease & Polak, 1978), and in medullary carcinoma of the thyroid (Shapiro *et al.*, 1985). These tumours show a common APUD (amine precursor uptake and decarboxylation) mechanism (Sisson *et al.*, 1981). Carcinoid tumours also possess a number of APUD properties, including in particular neurosecretory (dense core) granules and the ability to produce a variety of biogenic amines. Preliminary studies have suggested that carcinoid tumours may also take up $^{131}$I-MIBG (Sisson *et al.*, 1984; Smit *et al.*, 1984).

If carcinoid tumours were shown to take up this radiopharmaceutical with sufficient concentration then it might be possible to achieve therapeutic doses of $^{131}$I-MIBG, a technique successfully employed with malignant phaeochromocytoma (Sone *et al.*, 1985).

We describe a study to investigate further the uptake of $^{131}$I-MIBG for diagnostic imaging in 11 patients with confirmed metastatic carcinoid tumours.

Eleven patients known to have metastatic carcinoid tumours were studied. In 5 patients secondary deposits were histologically confirmed as carcinoid. The remaining 6 patients had metastatic tumours diagnosed by CT scanning or ultrasound and elevated urinary 5 hydroxyindoleacetic acid (5HIAA) levels, the original primary tumour had been diagnosed histologically. Ten of the 11 patients had abdominal CT scan or ultrasound to document metastases. An NMR scan was performed in one patient (10). Bone scans were only performed in patients with bone pain. Patient details are shown in Table I.

The patients received 0.3 ml of Lugol's iodine 3 times a day for 3 days prior to and 3 days after administration of $^{131}$I-MIBG to prevent thyroid uptake of the radioiodine.

$^{131}$I-MIBG (20–40 mBq) was injected intravenously. Imaging was performed at 24 and 48 h following injection for all patients, and at 72 h in 6 patients, using a Siemen's ZLC75 Gamma camera.

A medium energy collimator was used with the peak set at 350 KeV and a window of 15%. Images were acquired over 600 seconds. Planar anterior and posterior views of the chest and abdomen were taken with selective views of clinically involved areas.

**Table I** Patient characteristics.

| | | |
|---|---|---|
| Age | 41–72 | (Median 60) |
| Sex | M: 5 | F: 6 |
| No. of sites of disease | 1–3 | (Median 1) |
| 5HIAA levels | 115–1,198 | (Median 845) $\mu$mol 24 h$^{-1}$ |
| Sites of primary tumour | Ileum 4, Ovary 1, Lung 2<br>Unknown 4 | |

Correspondence: I.E. Smith.
Received 20 March 1988; and in revised form, 8 July 1988.

Uptake at tumour sites was compared with normal uptake both in the liver and the myocardium.

The levels of $^{131}$I-MIBG in the areas involved by carcinoid tumour in relation to the myocardial and liver uptakes are summarised in Table II together with the sites of disease, symptoms experienced and urinary 5HIAA levels for individual patients.

In 8 (73%) patients unequivocal abnormal uptake was seen. One patient (8) had abnormal uptake in a single area within the liver, whereas CT scan showed multiple metastases. One patient (2) demonstrated a mixture of 'hot' and 'cold' areas in the liver. In 3 patients no abnormal uptake was seen. No sites of disease were identified on $^{131}$I-MIBG scintigraphy that were not also detectable by other tests. One patient (5) with a negative $^{131}$I-MIBG scintigram was the only patient in our group to have bone metastases (histologically proven) and he also suffered no 'carcinoid' symptoms. A $^{99}$T$^{cm}$ methylenediphosphonate bone scintigram showed multiple areas of increased uptake, and this appearance was shown to be gradually progressing over a 4-year period. A second patient with a negative $^{131}$I-MIBG scintigram (11) had only moderately elevated 5HIAA levels and was asymptomatic. The third had a lung primary with hepatic metastases, an elevated 5HIAA and marked symptoms. The liver metastases were documented by ultrasound and CT scanning.

We found no correlation between the urinary 5HIAA levels and $^{131}$I-MIBG uptake in the tumours. One patient (6) had levels only modestly raised at 132 $\mu$mol 24 h$^{-1}$ ($N<75\,\mu$mol 24 h$^{-1}$) but an unequivocally positive scintigram and marked symptoms while another patient with 5HIAA levels 5 times this level had a negative scintigram.

We also found no relationship between 'carcinoid symptomatology' and $^{131}$I-MIBG uptake. Patient 5 suffered florid carcinoid symptoms (with elevated urinary 5HIAA levels) but no uptake of the $^{131}$I-MIBG was seen.

We did not observe an increase in uptake of the $^{131}$I-MIBG after 48 h and therefore scanning at 72 h was discontinued. Compared with other studies we saw low levels of myocardial activity at 48 h (none in 6/11 patients).

Our results confirm that $^{131}$I-MIBG is preferentially taken up by the majority of metastatic carcinoid tumours, with a 73% positive uptake in this study compared with 63% in Hoefnagel's series (Hoefnagel *et al.*, 1987). However, this incidence is lower than in malignant phaeochromocytoma where more than 90% of lesions take up $^{131}$I-MIBG (Wieland *et al.*, 1981). In keeping with other authors we found no correlation between urinary 5HIAA and $^{131}$I-MIBG uptake. However, we were unable to confirm the relationship between carcinoid symptomatology and $^{131}$I-MIBG uptake reported by Hoefnagel *et al.* (1987).

We were unable to demonstrate preferential uptake of radiolabelled MIBG in particular sites. Although our only patient with bony metastases did not show any uptake of $^{131}$I-MIBG, Hoefnagel found that 2 of 3 patients with bone involvement showed abnormal uptake.

In 2 patients the uptake varied from lesion to lesion. The $^{131}$I-MIBG scintigram suggested a solitary hepatic metastasis, while the CT scan showed multiple lesions. It is possible that these appearances represent a mixture of func-

**Table II**

| *Patient* | *Urinary 5HIAA* $N<75\,\mu mol\,24\,h^{-1}$ | *Sites* | *'Carcinoid' symptoms* | $^{131}$*I-MIBG uptake* | *Myocardial uptake* | *Confirmatory tests* |
|---|---|---|---|---|---|---|
| 1 | 845 | Li, P | D | Li=3, P=2 | 0 | CT Histo |
| 2 | 208 | Li | D | Li=3 and 0 | 0 | CT |
| 3 | 957 | Li | D, F, B | Li=3 | 1 | US |
| 4 | 986 | Li | Nil | Li=3 | 0 | CT Histo |
| 5 | 658 | Li | D, F, B | No abnormal uptake | 0 | US Histo |
| 6 | 132 | Li | D, F, B | Li=3 | 1 | CT |
| 7 | 115 | B | Nil | No abnormal uptake | 0 | XR Bone scan CT Histo |
| 8 | 1,198 | Li, S, N | D | Li=3, S=2, N=0 | 0 | CT US Histo |
| 9 | 517 | Li | F | Li=3 | 1 | US CT |
| 10 | 871 | Lu, M, N | D, F | Li=3, M=3[a] | 2 | CT NMR Histo |
| 11 | 177 | Li, Lu | Nil | No abnormal uptake | 1 | CT Histo |

[a]*N* obscured by liver.

Sites: Li = Liver, Lu = Lung, B = Bone, S = Spleen, N = Nodes, P = Pelvic mass, M = Mesentery

Symptoms: F = Flushing, D = Diarrhoea, B = Bronchospasm

Uptake grade:
0 No activity
1 Less than normal liver
2 Similar to normal liver
3 Greater than normal liver tioning and non-functioning lesions, or alternatively poorly functioning lesions may not be seen due to the low activities used and the limitations of planar imaging. This variability of uptake may have implications in future attempts at therapy.

The preferential uptake of $^{131}$I-MIBG by phaeochromocytomas has led to successful attempts at radiotherapeutic ablation using larger doses of this radiopharmaceutical (Sone *et al.*, 1985). A small number of patients with carcinoid have also been treated in this way and although some symptomatic improvements have been seen, no tumour regression has so far been reported (Hoefnagel *et al.*, 1987). Nevertheless the frequency of positive uptake suggests that the therapeutic potential merits further investigation.

### References

FISCHER, M., KAMANABROO, D., SONDERKAMP, H. & PROSKE, T. (1984). Scintigraphic imaging of carcinoid tumours with $^{131}$I-MIBG. *Lancet*, **ii**, 165.

HOEFNAGEL, C.A., DEN HARTOG JAGER, F.C.A., TAAL, B.G., ABELING, N.G.G.M. & ENGELSMAN, E.E. (1987). The role of $^{131}$I-MIBG in the diagnosis and therapy of carcinoids. *Eur. J. Nucl. Med.*, **13**, 187.

KIMMIG, B., BRANDEIS, W.E., EISENHUT, M., BUBECK, B., HERMANN, H.J. & ZUM WINKEL, K. (1984). Scintigraphic localisation of a neuroblastoma with $^{131}$I-MIBG. *J. Nucl. Med.*, **25**, 772.

PEASE, A.G.E. & POLAK, J.M. (1978). The diffuse neuroendocrine system and the APUD concept. In *Gut Hormones*, Bloom, S.R. & Grossman, M.I. (eds) p. 33. Churchill Livingstone: Edinburgh.

SHAPIRO, B., COPP, J.E., SISSON, J.C., EYRE, P.L., WALLIS, J. & BEIERWALTES, W.H. (1985). $^{131}$I-MIBG for the locating of suspected phaeochromocytoma: Experience in 400 cases. *J. Nucl. Med.*, **26**, 576.

SISSON, J.C., FRAGER, M.S., VALK, T.W. & 5 others (1981). Scintigraphic localisation of phaeochromocytoma. *New Engl J. Med.*, **305**, 12.

SISSON, J.C., SHAPIRO, B., BEIERWALTES, W.H. & 8 others (1984). Radiopharmaceutical treatment of malignant phaeochromocytoma. *J. Nucl. Med.*, **25**, 197.

SMIT, A.J., VAN ESSEN, C.H., HOLLEMA, H., MUSKIET, F.A.J. & PIERS, D.A. (1984). $^{131}$I-MIBG uptake in a non-secreting paraganglioma. *J. Nucl. Med.*, **25**, 984.

SONE, T., FUKUNAGA, M., OTSUKA, N. & 6 others (1985). Metastatic medullary thyroid cancer: Localisation with $^{131}$I-MIBG. *J. Nucl. Med.*, **26**, 604.

WIELAND, D.M., BROWN, L.E., TOBES, M.C. & 5 others (1981). Imaging the primate adrenal medulla with $^{123}$I and $^{131}$I-MIBG; concise communication. *J. Nucl. Med.*, **22**, 358.